# *Vital Signs*: Trends in Reported Vectorborne Disease Cases — United States and Territories, 2004–2016

**DOI:** 10.15585/mmwr.mm6717e1

**Published:** 2018-05-04

**Authors:** Ronald Rosenberg, Nicole P. Lindsey, Marc Fischer, Christopher J. Gregory, Alison F. Hinckley, Paul S. Mead, Gabriela Paz-Bailey, Stephen H. Waterman, Naomi A. Drexler, Gilbert J. Kersh, Holley Hooks, Susanna K. Partridge, Susanna N. Visser, Charles B. Beard, Lyle R. Petersen

**Affiliations:** 1 Division of Vector-Borne Diseases, National Center for Emerging and Zoonotic Infectious Diseases, CDC, Fort Collins, Colorado.

## Abstract

**Introduction:**

Vectorborne diseases are major causes of death and illness worldwide. In the United States, the most common vectorborne pathogens are transmitted by ticks or mosquitoes, including those causing Lyme disease; Rocky Mountain spotted fever; and West Nile, dengue, and Zika virus diseases. This report examines trends in occurrence of nationally reportable vectorborne diseases during 2004–2016.

**Methods:**

Data reported to the National Notifiable Diseases Surveillance System for 16 notifiable vectorborne diseases during 2004–2016 were analyzed; findings were tabulated by disease, vector type, location, and year.

**Results:**

A total 642,602 cases were reported. The number of annual reports of tickborne bacterial and protozoan diseases more than doubled during this period, from >22,000 in 2004 to >48,000 in 2016. Lyme disease accounted for 82% of all tickborne disease reports during 2004–2016. The occurrence of mosquitoborne diseases was marked by virus epidemics. Transmission in Puerto Rico, the U.S. Virgin Islands, and American Samoa accounted for most reports of dengue, chikungunya, and Zika virus diseases; West Nile virus was endemic, and periodically epidemic, in the continental United States.

**Conclusions and Implications for Public Health Practice:**

Vectorborne diseases are a large and growing public health problem in the United States, characterized by geographic specificity and frequent pathogen emergence and introduction. Differences in distribution and transmission dynamics of tickborne and mosquitoborne diseases are often rooted in biologic differences of the vectors. To effectively reduce transmission and respond to outbreaks will require major national improvement of surveillance, diagnostics, reporting, and vector control, as well as new tools, including vaccines.

## Introduction

Vectors are blood-feeding insects and ticks capable of transmitting pathogens between hosts. Wide varieties of pathogens have evolved to exploit vector transmission, including some viruses, bacteria, rickettsia, protozoa, and helminths. Dengue viruses are estimated to infect nearly 400 million persons worldwide each year (*1*), and malaria (*2*) is a major cause of pediatric mortality in equatorial Africa. Plague (*3*) and rickettsioses (*4*) cause deadly epidemics abroad. In the United States, 16 vectorborne diseases are reportable to state and territorial health departments, which are encouraged to report them to the National Notifiable Disease Surveillance System (NNDSS). Among the diseases on the list that are caused by indigenous pathogens are Lyme disease *(Borrelia burgdorferi*); West Nile, dengue and Zika virus diseases; plague (*Yersinia pestis*); and spotted fever rickettsioses (e.g., *Rickettsia rickettsii*). Malaria and yellow fever are no longer transmitted in the United States but have the potential to be reintroduced. As a group, vectorborne diseases in the United States are notable for their wide distribution and resistance to control. A Food and Drug Administration–approved vaccine is available to prevent only one of the notifiable diseases, yellow fever.

Despite the dissimilarities among vectorborne pathogens and the many vector species that can transmit them, commonalities exist. Vectorborne disease epidemiology is complex because of environmental influences on the biology and behavior of the vectors. The longevity, distribution, biting habits, and propagation of vectors, which ultimately affect the intensity of transmission, depend on environmental factors such as rainfall, temperature, and shelter. Most vectorborne pathogens are zoonoses, often with wild animal reservoirs, such as rodents or birds, making them difficult or impossible to eliminate. Arthropod vectors can bridge the gap between animals and humans that would not ordinarily intersect, as happens in Lyme disease, plague, and West Nile virus (WNV), facilitating the introduction of emerging animal pathogens to humans.

The pace of emergence of new or obscure vectorborne pathogens through introduction or belated recognition appears to be increasing. Since 2004, these have included two previously unknown, life-threatening tickborne RNA viruses, Heartland (*5*) and Bourbon (*6*), both reported from the U.S. Midwest. A tickborne relapsing fever agent, *Borrelia miyamotoi*, first described in Japan, has been found widely distributed in the United States (*7*) and another bacterial spirochete, *Borrelia mayonii* (*8*) was discovered in the upper U.S. Midwest. Two tickborne spotted fever *Rickettsiae*, *R. parkeri* (*9*) and *Rickettsia* species 364D *(**10**)*, and a tickborne *Ehrlichia* (*E. muris* *eauclairensis*) (*11*) were discovered to be pathogenic to humans. The mosquitoborne viruses chikungunya and Zika were introduced to Puerto Rico in 2014 and 2015, respectively. Zika virus is emblematic of the dangers of emergence. Zika was one of a number of obscure, mosquitoborne viruses known to be pathogenic to humans that are rarely encountered or studied (*12*). In the 60 years following its discovery in a monkey in Uganda, it was seldom reported as a human pathogen. In 2016, there were >36,000 cases reported in Puerto Rico, limited autochthonous, or local, transmission in Florida and Texas, and nearly 5,000 cases among travelers to the United States (*13*). The teratogenic consequences of the 2015–2017 epidemic in the region of the Americas were unexpected.

CDC examined trends of reported vectorborne disease cases in the United States during 2004–2016; this report discusses the challenges of prevention and control and highlights opportunities for vectorborne disease preparedness at the state and local level.

## Methods

Vectorborne disease data from NNDSS were retrieved from 2004, the first year that both neuroinvasive and nonneuroinvasive arthropodborne viral (arboviral) diseases were nationally notifiable, through 2016, the most recent year for which complete data are available (https://wwwn.cdc.gov/nndss/conditions/notifiable). Data were tabulated by disease, vector type (i.e., mosquito, tick, or flea), state or territory of residence, and year. State health departments report human disease cases using standard surveillance case definitions that include clinical and laboratory criteria. For some diseases, data reported according to Council of State and Territorial Epidemiologists definitions as confirmed or probable have been combined; autochthonous and travel-associated cases have been analyzed together by state or territory in which they were found.

Chikungunya virus, Zika virus, and *Babesia* cases became notifiable after 2004; only those data in NNDSS are presented. Although dengue became nationally notifiable only in 2010, earlier national data were available from CDC’s Dengue Branch and are included in this analysis.

## Results

**FIGURE 1:**
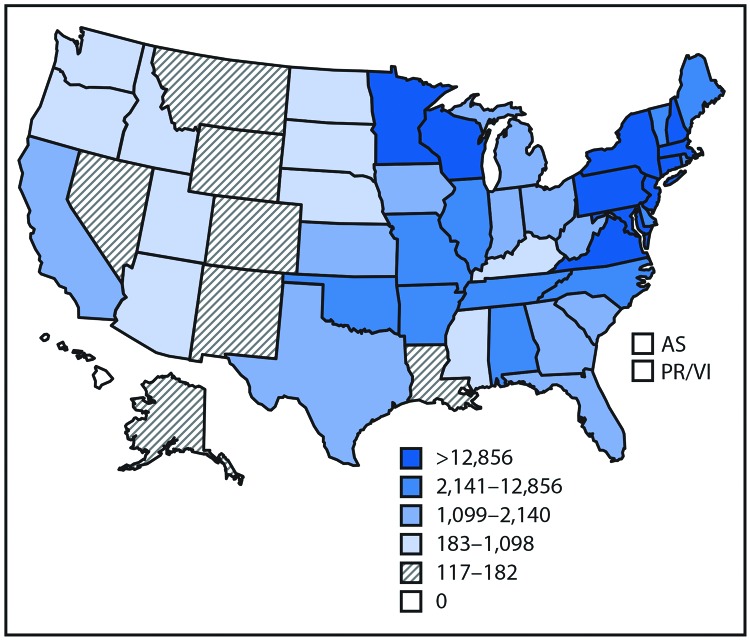
Reported cases* of tickborne disease — U.S. states and territories, 2004–2016 **Sources:** CDC, National Notifiable Diseases Surveillance System, 2016 Annual Tables of Infectious Disease Data. https://wwwn.cdc.gov/nndss/infectious-tables.html. CDC, Division of Health Informatics and Surveillance. CDC, ArboNET. **Abbreviations**: AS = American Samoa; PR/VI = Puerto Rico/U.S. Virgin Islands. * Data classified by quintile.

Nearly 650,000 cases of vectorborne disease were reported during 2004–2016 (Table). Tickborne diseases, which accounted for >75% of reports, occur throughout the continental United States, but predominate in the eastern part of the country and in areas along the Pacific Coast (Figure 1). Reported cases of tickborne disease have doubled in the 13-year analysis period, with Lyme disease accounting for 82% of cumulative reported tickborne disease. The combined incidence of reported anaplasmosis and ehrlichiosis, which are tickborne bacterial diseases, rose almost every year, as did spotted fever; babesiosis, a tickborne parasitic infection that has been notifiable since 2011, also contributed to the rise. Endemic plague, a fleaborne disease that is transmitted mostly in the rural southwestern United States, did not exceed 17 cases in a year. Tularemia and ehrlichiosis are geographically widespread but more prevalent in the central United States.

**TABLE:** Vectorborne disease cases reported to National Notifiable Disease Surveillance System — U.S. states and territories, 2004–2016*

Disease	Year													
	2004	2005	2006	2007	2008	2009	2010	2011	2012	2013	2014	2015	2016	Total
**Tickborne diseases**														
Lyme disease^†^	19,804	23,305	19,931	27,444	35,198	38,468	30,158	33,097	30,831	36,307	33,461	38,069	36,429	**402,502**
Anaplasmosis/Ehrlichiosis^§^	875	1,404	1,455	1,999	2,107	2,267	2,615	3,586	3,725	4,551	4,488	5,137	5,750	**39,959**
Spotted fever rickettsiosis^¶^	1,713	1,936	2,288	2,221	2,563	1,815	1,985	2,802	4,470	3,359	3,757	4,198	4,269	**37,376**
Babesiosis**	N	N	N	N	N	N	N	1,128	937	1,796	1,760	2,100	1,910	**9,631**
Tularemia	134	154	95	137	123	93	124	166	149	203	180	314	230	**2,102**
Powassan virus	1	1	1	7	2	6	8	16	7	15	8	7	22	**101**
**Subtotal tickborne diseases**	**22,527**	**26,800**	**23,770**	**31,808**	**39,993**	**42,649**	**34,890**	**40,795**	**40,119**	**46,231**	**43,654**	**49,825**	**48,610**	**491,671**
**Mosquitoborne diseases**														
Dengue viruses^††^	721	2,462	882	4,484	1,118	2,759	11,611	1,795	6,714	10,727	1,226	1,015	1,178	**46,692**
Zika virus	N	N	N	N	N	N	N	N	N	N	N	N	41,680	**41,680**
West Nile virus	2,539	3,000	4,269	3,630	1,356	720	1,021	712	5,674	2,469	2,205	2,175	2,149	**31,919**
Malaria**	1,458	1,498	1,476	1,411	1,257	1,456	1,778	1,726	1,504	1,594	1,654	1,397	1,958	**20,167**
Chikungunya virus	N	N	N	N	N	N	N	N	N	N	7,521	1,133	427	**9,081**
California serogroup viruses^§§^	118	80	69	55	62	55	75	137	81	112	96	70	53	**1,063**
St. Louis encephalitis virus	15	13	10	9	13	12	10	6	3	1	10	23	8	**133**
Eastern equine encephalitis virus	7	21	8	4	4	4	10	4	15	8	8	6	7	**106**
Yellow fever virus	0	0	0	0	0	0	0	0	0	0	0	0	1	**1**
**Subtotal mosquitoborne diseases**	**4,858**	**7,074**	**6,714**	**9,593**	**3,810**	**5,006**	**14,505**	**4,380**	**13,991**	**14,911**	**12,720**	**5,819**	**47,461**	**150,842**
**Fleaborne disease**														
Plague	3	8	17	7	3	8	2	3	4	4	10	16	4	**89**
**Total vectorborne diseases**	**27,388**	**33,882**	**30,501**	**41,408**	**43,806**	**47,663**	**49,397**	**45,178**	**54,114**	**61,146**	**56,384**	**55,660**	**96,075**	**642,602**

**Abbreviation:** N = not notifiable.

* U.S. territories included are Puerto Rico, U.S. Virgin Islands, and American Samoa.

^†^ Lyme disease reporting changed in 2008 to include probable cases in addition to confirmed cases.

^§^ Anaplasmosis and ehrlichiosis were reported separately after 2008 but are combined here for the entire period.

^¶^ Includes *R. rickettsii, R. parkeri, R.* species 364D.

** Surveillance data for babesiosis and malaria may be reported independently to different CDC programs; these data might vary slightly from those presented elsewhere.

^††^ Dengue became reportable to the National Notifiable Diseases Surveillance System in 2010. 2004–2009 data from Dengue Branch, Division of Vector-Borne Diseases, CDC.

^§§^ Includes Jamestown Canyon, La Crosse, and unspecified California serogroup viruses.

**FIGURE 2:**
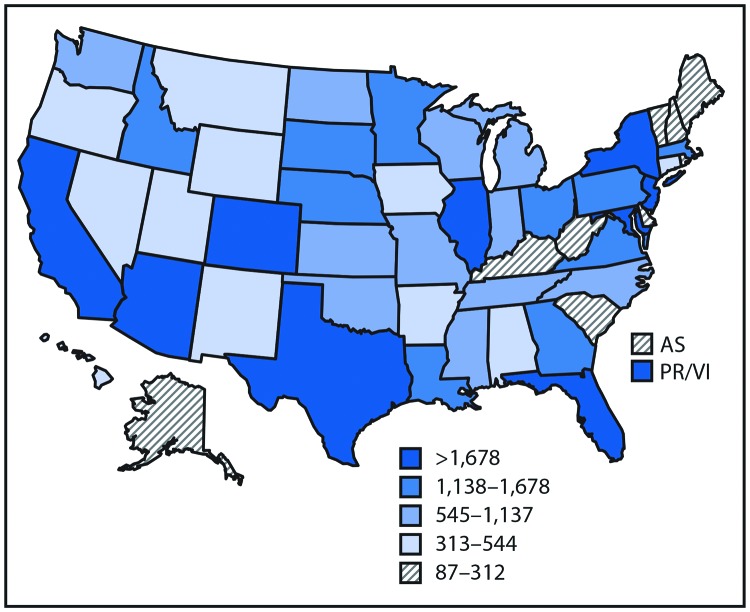
Reported cases* of mosquitoborne disease — U.S. states and territories, 2004–2016 **Sources:** CDC, National Notifiable Diseases Surveillance System, 2016 Annual Tables of Infectious Disease Data. https://wwwn.cdc.gov/nndss/infectious-tables.html. CDC, Division of Health Informatics and Surveillance. CDC, ArboNET. **Abbreviations**: AS = American Samoa; PR/VI = Puerto Rico/U.S. Virgin Islands. * Data classified by quintile.

**FIGURE 3:**
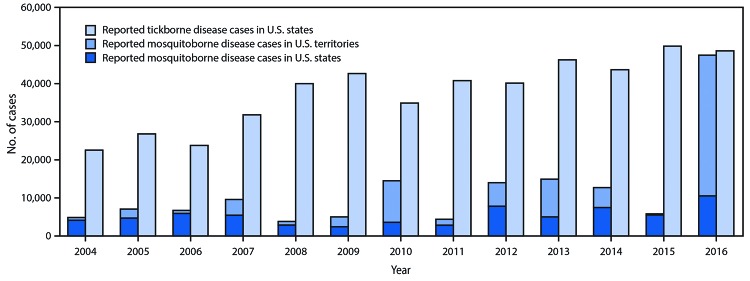
Reported nationally notifiable mosquitoborne,* tickborne, and fleaborne^†^ disease cases — U.S. states and territories, 2004–2016 * Mosquitoborne case counts include both locally transmitted and travel-associated cases. Only 305 arbovirus cases were reported from the territories in 2015. ^†^ A total of 89 fleaborne disease cases (plague) were reported during 2004–2018, ranging from two cases in 2010 to 16 cases in 2015. The cases are not depicted on the figure.

By contrast, the occurrence of mosquitoborne viruses was dispersed (Figure 2) and punctuated by epidemics (Table) (Figure 3). WNV was the most commonly transmitted mosquitoborne disease in the continental United States. Its most notable epidemic during 2004–2016 occurred in 2012, especially in Texas. Epidemics of dengue, chikungunya, and Zika viruses were mostly confined to the U.S. territories. All four dengue viruses were endemic in Puerto Rico, which was subject to cyclical epidemics, notably in 2010 and during 2012–2013. Puerto Rico’s first chikungunya virus epidemic peaked in 2014, followed by Zika virus in 2016. Travelers infected in the territories and Latin America accounted for >90% of the dengue, chikungunya, and Zika virus disease cases identified in the states and District of Columbia; limited autochthonous transmission of dengue occurred in Florida, Hawaii, and Texas, and of chikungunya and Zika viruses in Texas and Florida. Malaria is diagnosed in approximately 1,500 travelers yearly but no autochthonous transmission was documented during 2004–2016.

## Conclusions and Comments

These data indicate persistent, locality-specific risks and a rising threat from emerging vectorborne diseases, which have increasingly encumbered local and state health departments tasked with preventing, detecting, reporting, and controlling them. The overall case number masks two distinct trends. Epidemics characterize the mosquitoborne viruses. WNV transmission is effectively limited to the continental United States, whereas most dengue, chikungunya, and Zika virus transmission occurred in the territories. By contrast, the increasing reports of tickborne disease, which occurs almost exclusively in the continental United States, has been gradual. The area at risk for Lyme disease has been expanding (*14*). Although Lyme disease accounts for 82% of all reported tickborne diseases, spotted fevers, babesiosis, and anaplasmosis/ehrlichiosis have become increasingly prevalent. Diseases caused by pathogens that were relatively uncommon during the 13-year analysis period remain important because of their historical potential to cause epidemics (e.g., St. Louis encephalitis virus), their high case fatality rates (e.g., eastern equine encephalitis virus), or their potential as bioterror agents (e.g., plague and tularemia).

The reported data substantially underestimate disease occurrence. NNDSS relies on a person seeking care, a clinician requesting appropriate tests, and providers or laboratories reporting to public health authorities. Recent data from clinical and laboratory diagnoses estimate that Lyme disease infects approximately 300,000 Americans yearly, eight- to tenfold more than the number reported (*15*,*16*). Many arbovirus infections result in minimal symptoms. It has been estimated that 30–70 nonneuroinvasive arboviral disease cases occur for every WNV neuroinvasive disease case reported (*17*). Based on the number of neuroinvasive disease cases reported in 2016, between 39,300 and 91,700 nonneuroinvasive disease cases of WNV would have been expected to occur, but only 840 (1%–2%) were reported (*17*).

The dynamics of vectorborne pathogen transmission are significantly influenced by the characteristics of vector, reservoir, and host. Tickborne pathogens rarely cause sudden epidemics because humans are typically incidental hosts who do not transmit further, and tick mobility is mostly limited to that of its animal hosts. For ticks, the prolonged life cycle and widely separated blood feeds limit opportunities for pathogen transmission. *Ixodes scapularis*, for example, an important vector of *B. burgdorferi*, might feed on blood once in a year, but this is compensated for by their broad host preferences and the ability of single ticks to transmit multiple pathogen species. In contrast, the more mobile female mosquitoes feed on blood every 48–72 hours. Dengue, Zika, and chikungunya viruses are typically transmitted directly between humans by the mosquito, *Aedes aegypti*, after about a week’s extrinsic incubation period, resulting in explosive epidemics. WNV is one of the few purely zoonotic vectorborne pathogens with epidemic potential; humans are only at risk from mosquitoes that have fed on viremic birds. There must be a coincidence of flocks with a high prevalence of infection near humans when vector mosquito species are abundant. Bird movement was responsible for WNV’s rapid spread across the United States after its introduction to New York City in 1999.

The presence of competent vector species does not alone assure transmission. *Ae. aegypti*, whose range has been expanding, might now be present in up to 38 states (*18*), but despite the frequent arrival of travelers infected with dengue, chikungunya, or Zika viruses, autochthonous transmission has been rare. No local transmission of malaria resulted from the importation of about 1,500 cases annually, even though *Anopheles *mosquitoes are present in much of the United States. Although the range of *Ixodes scapularis* extends over much of the eastern United States, transmission of Lyme disease, *B. microti* babesiosis, and Powassan virus are rare outside of the Northeast and upper Midwest regions. Whatever the biologic, economic, behavioral, or land use reasons for these differences, the presence of vectors with proven or possible capacity to transmit a wide range of pathogens leaves the United States susceptible to outbreaks of exotic vectorborne diseases, as demonstrated by the limited local transmission of dengue and Zika viruses in Florida and Texas.

The findings in this report are subject to at least three limitations. First, underreporting might have substantially limited the number of cases analyzed. As noted, the number of Lyme disease cases reported to NNDSS is estimated to represent a fraction of incident cases. In addition, because many patients with dengue, nonneuroinvasive West Nile, and Zika virus infections experience mild symptoms, they might not seek medical attention. Second, not all the diseases described in this report were reportable for the full 13-year analysis period or from all states and territories; babesiosis data are only available from 2011 from some states. Finally, although CDC collected national dengue data before 2011, the first year it was officially designated as notifiable, it is possible a higher proportion of cases were reported after reporting became mandatory. Overall, it is likely the actual number of vectorborne disease cases substantially exceeds those described in this report.

In the face of increasing incidence and threat from novel pathogens, the burden on local and state public health departments has increased. Critical to effectively preventing or responding to disease outbreaks is sensitive disease and vector surveillance, backed by well-organized, well-prepared, and sustained vector control operations. Good surveillance and reporting depend on rapid, accurate diagnostic confirmation; more sensitive and specific tests that can be used locally are needed. Vaccines against Lyme disease, dengue, chikungunya, and Zika, goals of intense research and development, could reduce risk from those major threats. The tools for vector control are limited but can be effective when implemented rapidly. Ticks have been especially difficult to control (*19*), increasing the responsibility for personal protective measures. Nearly all public vector control operations in the United States are locally funded and operated. Networks of vector control operatives are essential to support threat reduction and counter outbreaks, yet in a recent national survey 84% of 1,083 local mosquito control organizations reported lacking one or more of five core vector control competencies (*20*). Resources available to assist state and local health departments could be used to develop vector control program competencies.

Reducing vectorborne disease incidence and responding to outbreaks is a large and complex challenge. CDC is using two strategies to mitigate vectorborne threats: advancing innovation and discovery and rebuilding comprehensive vector control programs that have eroded over time (*20*). CDC works with states, territories, and tribal councils to compile surveillance data, develop strategies and guidance, and educate the public about specific threats and prevention measures for populations at risk. Expanding sustainable vectorborne disease prevention programs is needed to respond to the ongoing and increasing threat of vectorborne disease.

**Key Points**

• A total of 642,602 cases of 16 diseases caused by bacteria, viruses, or parasites transmitted through the bites of mosquitoes, ticks, or fleas were reported to CDC during 2004–2016. Indications are that cases were substantially underreported.

• Tickborne diseases more than doubled in 13 years and were 77% of all vectorborne disease reports. Lyme disease accounted for 82% of all tickborne cases, but spotted fever rickettsioses, babesiosis, and anaplasmosis/ehrlichiosis cases also increased.

• Tickborne disease cases predominated in the eastern continental United States and areas along the Pacific coast. Mosquitoborne dengue, chikungunya, and Zika viruses were almost exclusively transmitted in Puerto Rico, American Samoa, and the U.S. Virgin Islands, where they were periodically epidemic. West Nile virus, also occasionally epidemic, was widely distributed in the continental United States, where it is the major mosquitoborne disease.

• During 2004–2016, nine vectorborne human diseases were reported for the first time from the United States and U.S. territories. The discovery or introduction of novel vectorborne agents will be a continuing threat.

• Vectorborne diseases have been difficult to prevent and control. A Food and Drug Administration–-approved vaccine is available only for yellow fever virus. Many of the vectorborne diseases, including Lyme disease and West Nile virus, have animal reservoirs. Insecticide resistance is widespread and increasing.

• Preventing and responding to vectorborne disease outbreaks are high priorities for CDC and will require additional capacity at state and local levels for tracking, diagnosing, and reporting cases; controlling vectors; and preventing transmission.

• Additional information is available at https://www.cdc.gov/vitalsigns/.
